# Full-field stimulus threshold testing: a scoping review of current practice

**DOI:** 10.1038/s41433-023-02636-3

**Published:** 2023-07-13

**Authors:** Linda F. Shi, Amanda J. Hall, Dorothy A. Thompson

**Affiliations:** 1 Tony Kriss Visual Electrophysiology Unit, Clinical and Academic Department of Ophthalmology, Great Ormond Street Hospital for Children NHS Foundation Trust, London, UK; 2 College of Health and Life Sciences, Aston University, Birmingham, UK; 3 UCL Great Ormond Street Institute for Child Health, University College London, London, UK

**Keywords:** Diagnosis, Outcomes research

## Abstract

The full-field stimulus threshold (FST) is a psychophysical measure of whole-field retinal light sensitivity. It can assess residual visual function in patients with severe retinal disease and is increasingly being adopted as an endpoint in clinical trials. FST applications in routine ophthalmology clinics are also growing, but as yet there is no formalised standard guidance for measuring FST. This scoping review explored current variability in FST conduct and reporting, with an aim to inform further evidence synthesis and consensus guidance. A comprehensive electronic search and review of the literature was carried out according to the Preferred Reporting Items for Systematic Reviews and Meta-analysis Extension for Scoping Reviews (PRISMA-ScR) checklist. Key source, participant, methodology and outcomes data from 85 included sources were qualitatively and quantitatively compared and summarised. Data from 85 sources highlight how the variability and insufficient reporting of FST methodology, including parameters such as units of flash luminance, colour, duration, test strategy and dark adaptation, can hinder comparison and interpretation of clinical significance across centres. The review also highlights an unmet need for paediatric-specific considerations for test optimisation. Further evidence synthesis, empirical research or structured panel consultation may be required to establish coherent standardised guidance on FST methodology and context or condition dependent modifications. Consistent reporting of core elements, most crucially the flash luminance equivalence to 0 dB reference level is a first step. The development of criteria for quality assurance, calibration and age-appropriate reference data generation may further strengthen rigour of measurement.

## Abstract (zh)

全视野刺激阈值(FST)是测量全视野视网膜光敏感性的心理物理指标。它可以评估严重视网膜疾病患者的残余视功能, 并越来越多作为临床试验的终点。FST在临床的应用也不断增加, 但是目前还没有正式的关于FST测量标准的指南。本综述探讨了目前FST开展和报告的差异性, 旨在为进一步证据分析和指导共识提供信息。根据系统分析与综述和PRSIMA-ScR检查表对文献进行了全面的电子检索与回顾。对纳入的85个关键来源、参与者、方法和结果数据进行定性和定量对比与总结。来自85个来源的数据强调了FST方法的可变性和报告的不足之处, 包括闪光亮度, 颜色, 持续时间, 测试方法和暗适应等参数, 这些因素可能阻碍了各中心对于其临床意义的比较和解释。本综述也强调了在测试优化方面, 尚未满足对儿科特定考虑的需求。可能需要进一步的综合证据、实证研究或结构化小组进行商讨, 以建立FST方法和环境或条件依赖性修改的连续性标准化的指导。关键的一致报告, 将最重要的闪光灯亮度设置于0dB参考水平是第一步。制定质量保证、校准和适龄参考数据生成的标准可进一步加强测量的严谨性。

## Introduction

The full-field stimulus threshold test (FST) is a psychophysical measure of whole-field retinal light sensitivity. It was developed as an alternative method to assay residual visual function in patients with inherited retinal disease (IRD), whose severely reduced vision may prohibit standard quantitative follow-up with LogMAR acuity, perimetry and electroretinography (ERG) [1, 2]. In the recent decade as clinical trials for genetic therapies in IRDs have begun to advance into real world applicability, FST garnered momentum as a research outcome measure, and now increasingly as an adjunctive method for the assessment and surveillance of low vision patients in the clinical setting [3, 4].

In brief, the test stimulus is a full-field (‘Ganzfeld’) flash or pulse of light often generated using narrow-band light emitting diodes (LEDs). The participant responds ‘seen’ or ‘not seen’ to stimuli of varying luminance strengths. The stimulus luminance increases or decreases strategically until sampling is sufficient to calculate an estimate of the light perception threshold using a psychometric function. Responses to chromatic (red/blue/green) or achromatic (white) stimuli, presented on a variety of light- or dark-adapted backgrounds, are used to delineate the relative contributions of cone and rod photoreceptors to the sensitivity threshold estimate [1, 3]. Current commercially available FST equipment include the Diagnosys*FST* programme with the Espion ColorDome™ (Diagnosys LLC, Lowell, MA, USA) and the Metrovision FST programme on the MonCvONE perimeter (Metrovision, Pérenchies, France).

### FST in gene therapy trials and wider research applications

Originally FST was a secondary efficacy measure in gene therapy clinical trials for voretigene neparvovec-rzyl *(*VN), now approved in several global territories for *RPE65*-mediated retinopathy [5, 6]. This early-onset retinal dystrophy (also called Leber’s congenital amaurosis, LCA2) is characterised by severe progressive visual impairment and rod-cone degeneration from infancy [7, 8].

Early phase VN publications mention FST with chromatic stimuli as a functional outcome measure [9, 10]. The later Phase III and long-term safety and efficacy studies report both chromatic and achromatic FST, with the change in white light FST threshold averaged across both eyes reported as a secondary efficacy outcome [11–13]. The primary endpoint in these *RPE65* clinical trials remained an improvement in the multi-luminance mobility test (MLMT). Nonetheless, a strong correlation was shown between 1-year change in MLMT and the white light FST response averaged over both eyes (Pearson correlation coefficient r = 0.71), ratifying FST as a feasible surrogate measure where mobility testing may be unavailable [4, 12]. Currently, an active clinical trial in Japanese *RPE65* retinal dystrophy patients lists 1-year change in FST as its primary clinical end point (NCT04516369).

The success of the VN trials saw uptake of FST as an outcome measure in other clinical trials for IRDs, including subretinal *hMERTK* therapy for patients with advanced retinitis pigmentosa (RP) (NCT01482195) (ref. [14]), electronic retinal prosthesis for end-stage RP (NCT02720640) (ref. [15]), and intravitreal antisense oligonucleotide therapy in *CEP290-*LCA (LCA10; NCT03140969) (ref. [16, 17]). Increasingly FST is being included among the battery of visual function markers in various phenotyping studies, either in anticipation of potential future therapy development, or to monitor natural history of visual decline. These include *TRPM1*-congenital stationary night blindness [18], *CRB1*-RP [19, 20], *CNM4* Jalili syndrome [21], retinal degeneration in Usher Syndrome ([22]; NCT05158296; NCT04765345), and other forms of LCA including *GUCY2D-*LCA (LCA1) (ref. [23]) and *AIPL1*-LCA (LCA4) (ref. [24]). FST has also been used as an alternative to dark adaptometry to phenotype dark adaptation in choroideremia [25, 26], and to quantify cone sensitivity during the cone plateau in Stargardt disease [27]. A further interesting role has been proposed for FST to be a measure of rod inhibition for visual cycle-modifying drugs that block *RPE65* function [28, 29].

A given drawback of the full-field nature of FST is the inability to localise threshold improvements at specific retinal loci. As such, the retinal origin of the FST response remains contested. Based on initial comparisons against dark-adapted perimetry, FST responses are commonly assumed to originate from the most sensitive retinal areas [1]. This appeared to be corroborated by another study that found FST blue and white thresholds were inversely correlated with maximum dark-adapted perimetric sensitivity (r = –0.8) at low luminance, though the correlation weakened at higher luminance where thresholds were likely to be influenced by cones [30]. Conversely, other studies correlating FST thresholds with ERG and microperimetry have suggested responses may be generated within the central 20 degrees of the visual field [31], or mediated by spatial summation [32].

### FST as a clinical tool

Given the scaling challenges of using MLMT in a clinical setting, FST has become a favoured clinical surrogate follow-up measure of post-treatment efficacy for *RPE65* patients [33, 34]. Additionally, FST may be clinically useful for monitoring patients with early-stage retinal disease [22], or as an auxiliary test for patients with low vision and poor central fixation [35]. However, without harmonised reference data or best-practice consensus for FST, protocol variations between institutions currently limit data comparability and interpretation of quality.

Clinical practice guidelines often require that tests are adapted to become feasible and practical for specialised populations [36]. The specific utility of FST in IRD means the clinical population will invariably include children and/or those with complex sensory and developmental needs. Stingl’s group [37] reported a strong correlation between age and three-month FST improvement to blue stimuli in treated *RPE65* patients (regression analysis R^2^ = 0.81), suggesting a higher chance of rescuing rod function with treatment at a younger age. Currently the evidence base for FST protocol modification is unclear, and specific reference data are lacking for paediatric and more specialised populations.

### Scoping review aims & objectives

Ahead of the development and formalisation of standardised clinical practice guidance for FST, it will be valuable to understand the scope of practice of how the FST is delivered and reported in different contexts, compare the methodological variability of testing protocols, and identify potential areas for adaptation or optimisation for specific populations. As summative research about FST is limited a scoping review was chosen to systematically survey the current available literature on the FST, with the hope that findings may form the basis for future systematic review and evidence synthesis.

## Methods

The scoping review is reported according to the Preferred Reporting Items for Systematic Reviews and Meta-analysis Extension for Scoping Reviews (PRISMA-ScR) checklist, and methodology followed guidance from the Joanna Briggs Institute (JBI) [38]. The research question was to explore FST practice in human participants in research and clinical settings to date. A comprehensive electronic Boolean search was conducted in the Cochrane Library, EMBASE, Pubmed, Scopus and Web of Science, using keyword combinations based on ‘FST’, ‘full-field’, ‘stimulus’, ‘sensitivity’, ‘scotopic’, ‘threshold’, ‘retina’, ‘night vision’ etc, with search refinement guided by a library information specialist (C.L., Aston University Library). The full search query is listed in Appendix Table 1. Electronic databases were last searched in September 2022.

To minimise publication bias and maximise scope, grey sources were located manually from bibliographies of key studies or by searching grey literature repositories and clinical trial registries using selected keyword combinations. Grey literature also included educational and technical documents such as user manuals and commercial symposia presentations, sourced through Google searching or obtained directly from the manufacturer.

All identified citations were collated into Mendeley Desktop Version 1.19.4 (Elsevier, London, UK). Following duplicates removal, title and abstracts of references were screened before selected texts were retrieved and assessed in full against the inclusion criteria (Appendix Table 2). The process was checked by a second reviewer (A.H.) and any discrepancies resolved through discussion. Included items were all sources where full-field luminance stimuli were used to quantify visual sensitivity thresholds in human participants using a psychometric method. Items were excluded if they were in a non-human setting, if there was no appreciable reporting of FST methodology or outcomes, or if the full text was not available.

Data extracted included key characteristics regarding source, participant, test and key results and recorded in a data extraction form in Microsoft Excel. Categories and subcategories of the full data extraction form are listed in Appendix Table 3. Data extraction from non-English language papers was aided by free online translation platforms where appropriate. A web-based application was used to derive numerical data estimates from figures or graphs where relevant (WebPlotDigitizer, https://apps.automeris.io/wpd/).

To present a narrative overview of all sources included, data relating to key source characteristics, participant characteristics, test characteristics and key outcomes were counted and summarised. Concepts were categorised and coded to explore relationships among common themes and all data visualisation was performed using Microsoft Office.

## Results

### Search and selection of data

**Fig. 1:**
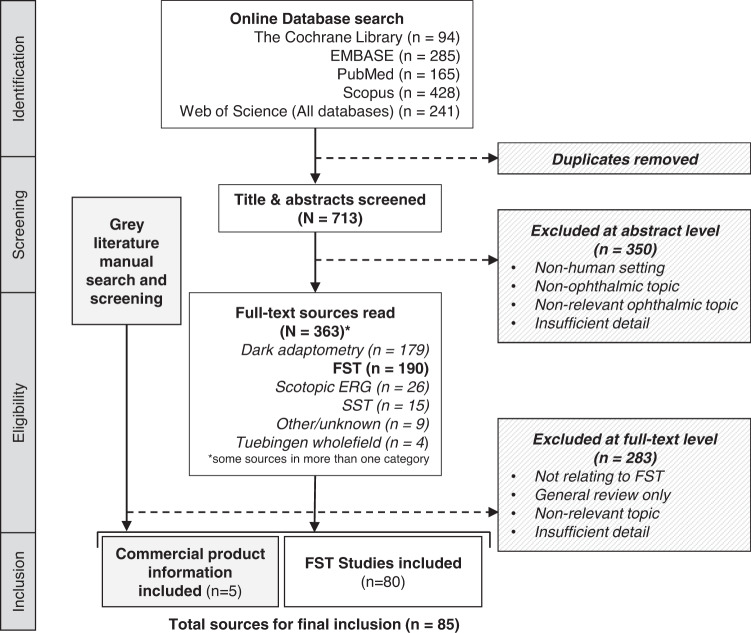
Scoping review flow diagram. PRISMA-ScR flow diagram of source selection process (from Tricco et al., 2018).

The full study selection process is presented as a PRISMA-ScR flow diagram [39] (Fig. 1). After removal of duplicates, 713 unique abstracts were screened against inclusion criteria. First stage screening was checked in parallel by a second reviewer (A.H.) using a randomly selected sample of titles and abstracts and any disagreement was resolved through discussion. In total, 363 items from screening were retrieved in full for further assessment alongside manually sourced grey literature.

The broad search initially sought to capture analogous modalities or techniques to FST, such as the whole-field Scotopic Sensitivity Tester (SST-1) (LKC Technologies, Gaithersburg, MD, USA) which is no longer commercially available, or studies deriving the scotopic final threshold from the dark adaptometry curve. For improved feasibility and relevance of the scope it was agreed with other authors (A.H. and D.T.) that data extraction and synthesis should focus on items pertaining to the current commercially available versions of the FST. 85 total sources were eligible for final inclusion [1–4, 9–12, 14–27, 29–35, 37, 40–94], comprising published studies using FST (*n* = 80) and product information available from manufacturers (*n* = 5) (Appendix Table 4). Quantitative summarisation and evidence synthesis will refer to these either separately as ‘FST studies’ and ‘commercial information’, or collectively as ‘sources’. The findings from evidence synthesis are reported under the four broad themes of ‘Scope’, ‘Population and context’, ‘Methodology and reporting’, and ‘Interpretation’.

### Scope of FST sources

**Fig. 2:**
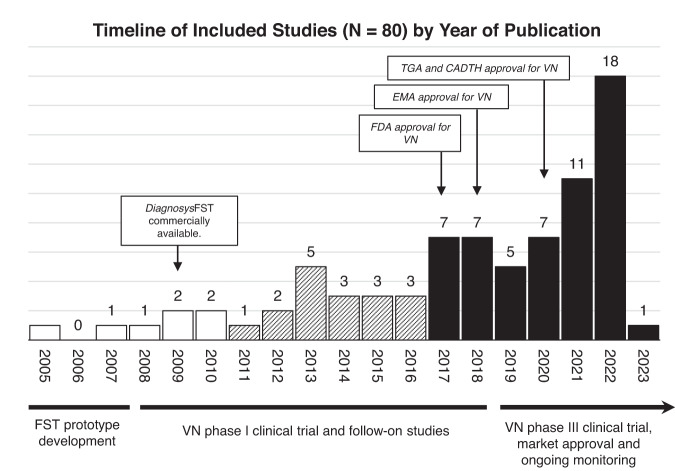
Summary figure showing timeline of included sources (*n* = 80) by year of publication, with key stages and publications in full-field stimulus test (FST) development and voretigene neparvovec (VN) clinical trial progression and approval. CADTH Canadian Agency for Drugs and Technologies in Health, EMA European Medicines Agency, FDA Food and Drug Administration, TGA Therapeutic Goods Administration.

The general publication characteristics of the included FST sources are summarised in Table 1. Of the 85 total sources available since 2005, the majority (72%) of items are from the most recent five years to date, reflecting escalating interest in FST as an outcome measure after its inclusion in the landmark *RPE65* clinical trials (Fig. 2). There was high variability and inconsistency in author self-reporting of study design type, and studies were re-classified for this review based on published definitions [95]. Most included studies were retrospective evaluations of a specific IRD phenotypes (*n* = 12), longitudinal or natural history studies (*n* = 11), or explorations/comparison of novel outcome measures in the specified IRD population (*n* = 8)

**Table 1:** Summary of source characteristics.

Source characteristics	Number (*N* = 85 total)	Percentage
Publication year		
2005–2010	7	8%
2011–2016	17	20%
2017–2023	61	72%
Source type		
Interventional/clinical trials		
Phase I/II	6	
Phase III	3	
Other	6	
Prospective trial	(3)	
Dose escalation trial	(1)	
Post-hoc analysis	(1)	
Mixed (phenotype evaluation and interventional trial)	(1)	
Observational studies (based on Bhopal, 2016)		
Descriptive (case report, case series, phenotype evaluation, retrospective chart review)	18	
Cohort (prospective, longitudinal/natural history)	13	
Case-control	–	
Cross-sectional	–	
Mixed methods (Cross sectional and longitudinal)	3	
Conference proceedings		
Abstract	14	
Poster	1	
Other		
Exploratory evaluation or comparison of outcome measures	9	
Review	4	
Proof-of-concept/feasibility	3	
Commercial/manufacturer materials	5

**Fig. 3:**
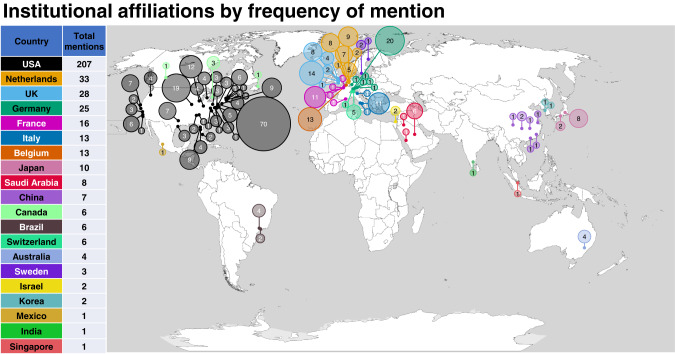
Geographic infographic of institutional affiliations of all included sources (*n* = 85) by country and city (or USA state). Area of circle is proportional to the total frequency count of mentions for the institution as listed within the Author Affiliations across all sources.

Institutional affiliations spanned 20 countries (Fig. 3), with the United States (USA) having the highest number of mentions in included sources (207 mentions) followed by the Netherlands (33 mentions). Specifically in the USA, institutions from Philadelphia were the most frequently mentioned (68 mentions) in author affiliations. This may be consistent with these being sites for the groups involved in the preclinical, clinical and commercialisation work for VN gene therapy [13, 40] and the development of the initial and current iterations of the FST [1, 2, 4].

There were no included sources affiliated with sites based in the continent of Africa. Two groups in Brazil [31, 41–43] represent the only mentioned centres in South America. There were four publications affiliated with two institutions in Sydney. This high representation of published research from North America and Western Europe may reflect the geographic distribution of centres with specialised interest and resource available for genetic eye disease and vision science research. There may also be limitations of the search strategy used in this review, despite effort to search non-English language databases. Moreover, this could also highlight a lack of resourcing, infrastructure and funding for carrying out specialised ophthalmic testing or clinical trials in these lesser represented regions

### Population and context

The commercial product information sources (*n* = 5) carry no relevant patient population data so are omitted from this part of quantitative synthesis and used for reference purposes where appropriate.

#### Clinical genotype and phenotype

Study populations of included FST studies (*n* = 80) are mainly patients with IRD and/or visually healthy participants used as controls. This is consistent with the FST having been initially developed expressly for this low vision patient population [1]. Non-IRD populations in which FST has been performed includes diabetic retinopathy [41, 44], age-related macular degeneration [1, 45] and one study in only visually healthy controls [46].

Broadly, in rod and rod-cone retinal dystrophies, the rationale for using FST has been to establish or characterise residual rod photoreceptor function [23], measure natural history of rod-cone functional decline [22], or as a supplementary measure of visual function changes e.g. after gene supplementation [17, 34, 96]. In macular or cone dystrophy populations, FST has been used as an alternative measure of dark-adapted thresholds for patients who have difficulty maintaining the fixation necessary to perform dark-adapted perimetry [27], or used with different chromatic stimuli and backgrounds to interrogate differential photoreceptor sensitivity [47].

**Fig. 4:**
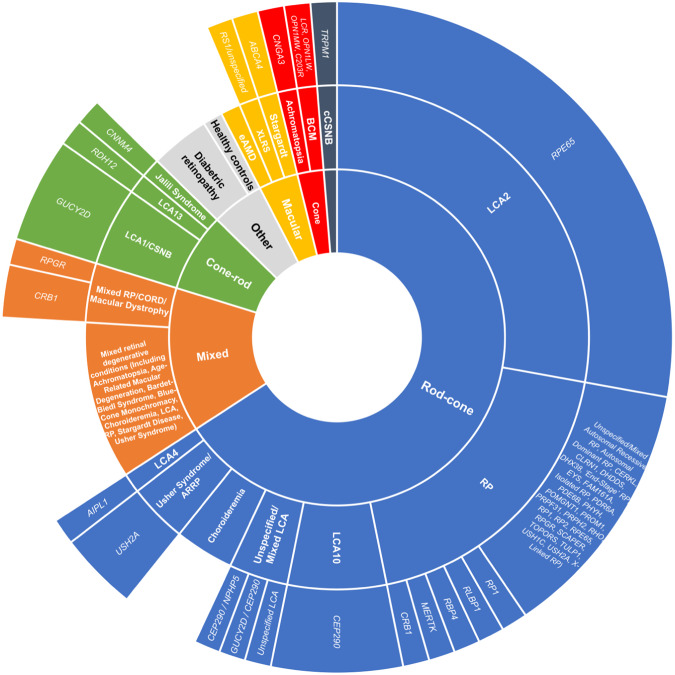
Patient population of included studies (*n* = 79) categorised by pattern of retinal dysfunction, clinical phenotype and genotype. The area of each segment is proportional to the number of FST studies with this clinical population. One study omitted from figure due to being a narrative review. BBS Bardet-Biedl syndrome, BCM blue-cone monochromacy, CORD cone-rod dystrophy, cCSNB complete congenital stationary night blindness, eAMD exudative age-related macular degeneration, LCA Leber congenital amaurosis, RP retinitis pigmentosa, XLRS X-linked retinoschisis.

The study population data of included studies are tabulated in Table 2 and represented in Fig. 4, categorised by pattern of retinal abnormality, followed by clinical phenotype and genotype, although some studies had mixed or unspecified populations.

**Table 2:** Studies categorised by phenotype and/or genotype.

Primary study population by phenotype and genotype	Number of studies (Total *N* = 80)	Includes visually healthy controls
Leber Congenital Amaurosis/Early Onset Retinal Dystrophy (LCA/EORD)		
* RPE65*	22	1
* CEP290*	5	1
* GUCY2D*	4	–
* CEP290 / NPHP5*	1	1
* GUCY2D / CEP290*	1	1
* AIPL1*	1	–
* RDH12*	1	1
Unspecified/mixed LCA	1	–
Retinitis pigmentosa (RP)		
* USH2A*	3	–
* CRB1*	1	1
MERTK	1	–
* RBP4*	1	–
* RLBP1*	1	–
* RP1*	1	–
Unspecified/Mixed RP (Including Mixed Populations of *Autosomal Recessive RP, Autosomal Dominant RP, CERKL, CLRN1, DHDDS, DHX38, ‘End-Stage’ RP, EYS, FAM161A, Isolated RP, PDR6A, PDE6B, PHYH, POMGNT1, PROM1, PRPF31, PRPH2, RHO, RP1, RP2, RPE65, RPGR, SCAPER, TOPORS, TULP1, USH1C, USH2A, X-Linked RP*)	10	2
Mixed Retinal Degenerative Conditions (Including Achromatopsia, Age-Related Macular Degeneration, Bardet-Biedl Syndrome, Blue-Cone Monochromacy, Choroideremia, LCA, RP, Stargardt Disease, Usher Syndrome)	8	5
Mixed RP/CORD/Macular Dystrophy		
* CRB1*	2	–
* RPGR*	1	–
Choroideremia	3	3
Diabetic Retinopathy (DR)		
Proliferative DR	2	1
Non-Proliferative DR	1	–
Blue-Cone Monochromacy		
* LCR, OPN1LW, OPN1MW, C203R*	1	–
Achromatopsia		
* CNGA3*	1	–
Complete Congenital Stationary Night Blindness		
* TRPM1*	1	–
Exudative Age-Related Macular Degeneration	1	1
Healthy Controls Only	1	1
Jalili Syndrome		
* CNNM4*	1	–
X-Linked Retinoschisis		
* RS1/Unspecified*	1	1
Stargardt Disease		
* ABCA4*	1	1
Not Applicable (Review)	1	–

#### FST and patient age

There were nine studies with no available age data. Of the other 71 studies, the age of the total study patient population ranged from 6 months to 87 years old at baseline or time of reporting. 44 of these studies (62%) had a study population that overall included patients aged <18 years, of which 15 studies included children aged ≤5 years (pre-school age). FST was not always achieved nor attempted in all patients in the total study population, with reasons given including patient reported as having no or minimal light perception [48, 49], unavailability of equipment [40, 50, 51], adverse complications [52] or age-related reasons. Specifically reported reasons for not achieving FST in patients of younger age include ‘exhaustion’ [12], results being ‘too variable’ [35], unreliable performance [11] or simply ‘young age’ [20].

**Fig. 5:**
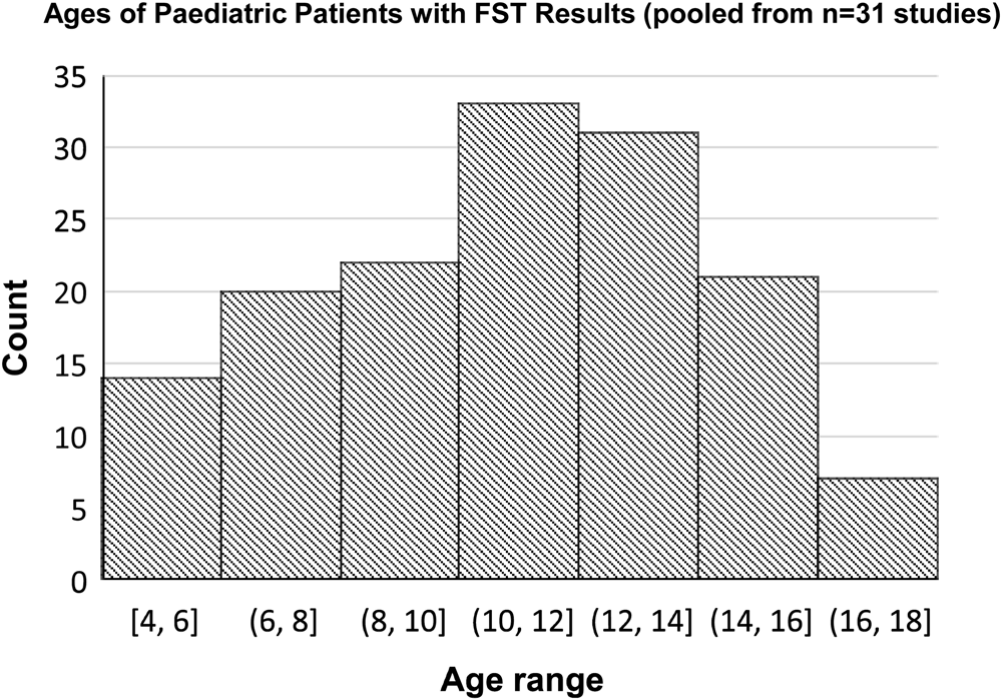
Ages of individual paediatric patients reported to have achieved results from FST testing (*n* = 148 children pooled from 31 studies where individual ages were reported). Median age of these children is 11 (Interquartile range 9–14).

For eleven of the 44 studies that included children in their total population, FST was not reported on an individual patient basis and only as a summative statistic so it was not possible to know if FST results were specifically achieved in those under 18 years of age. This was usually due to the study population being large [1, 3, 53, 54] or the source being a conference abstract [55]. The 44 studies are tabulated in Table 3 showing overall age range, number of patients with reportable FST thresholds, and ages of individual children in whom FST results were achieved. Age data from the final column of Table 3 of 148 children from 31 studies are pooled and plotted as a histogram (Fig. 5), with median age 11 and IQR 9–14 years.

**Table 3:** Characteristics of studies with paediatric populations (*n* = 44), ordered by overall study population age range (youngest to oldest).

Source	Clinical population	Overall study population age range (Years)	Total number of patients with reportable FST results (total study population)	Ages of individual paediatric patients with reportable FST results (Years)
Jacobson et al., 2013 [82]	*GUYCY2D-*LCA	6 months–37	9 (11)	7, 7, 11, 13
Jacobson et al., 2011 [24]	*AIPL1*-LCA	6 months–45	5 (11)	16
Banin et al., 2010 [72]	*RPE65*-LCA	1–64	2 (29)	No patients < 18 achieved FST
Aleman et al., 2018 [35]	*RDH12* mutations	2–17	18 (29)	6, 6, 7, 8, 8, 9, 9, 10, 10, 11, 11, 11, 11, 11, 13, 13, 17
Sengillo et al., 2022 [54]	*RPE65*	2–44	12 (41)	’10 patients under 20’
Jacobson et al., 2017 [83]	*GUYCY2D-*LCA	2–59	24 (28)	7, 7, 7, 9, 11, 11, 12, 12, 13, 13, 14, 14
Deng et al., 2022 [34]	Biallelic *RPE65* mutations	4-17	7 (13)	Age not individually reported
Ku et al., 2021 [55]	*RPE65* retinopathy	4–38	6 (11)	Not individually reported (abstract)
Russell et al., 2017 [11]	Biallelic *RPE65* mutations, Luxturna trial Ph III	4–44	29 (30)*	4, 5*, 5, 5, 5, 6, 6, 6, 6, 7, 8, 9, 9, 10, 11, 11, 11, 13, 13, 16
Maguire et al., 2019 [12]	Biallelic *RPE65* mutations, Luxturna trial Ph I & III	4–44	37 (40)	Not individually reported
Hyde et al., 2021 [21]	*CNNM4* Jalili syndrome	5–15	2 (3)	14, 15
Gange et al., 2022 [53]	*RPE65*-LCA patients with postoperative perifoveal chorioretinal atrophy	5–20	Not reported (10)	Not individually reported
Jacobson et al., 2017 [49]	*CEP290*-LCA	5–48	14 (22)^†^	10, 11, 12, 14, 15
Luo et al., 2015 [47]	Blue Cone Monochromacy	5–72	15 (25)	5, 7, 10, 12, 13, 14, 16
Klein & Birch, 2009 [3]	Retinal degenerative disease (ADRP, ARRP, isolated RP, XLRP, LCA, Usher syndrome, BBS, CORD)	5–84 23-52 healthy control	42 (42) patients 7(7) healthy control	Not individually reported
Bedoukian et al., 2022 [69]	*RP1* recessive RP	6	1 (1)	6
Talib et al., 2021 [59]	*CRB1* retinal dystrophy	6–74	15 (22)	6^‡^, 10^‡^, 11, 12
Roman et al., 2022 [4]	Inherited retinal disease (LCA, RP, Usher, CORD, BBS, choroideremia, BCM, achromatopsia, Stargardt)	6–87	273 (273)	11, 13, 15, others not individual reported
Nguyen et al., 2022 [20]	*CRB1* retinal dystrophy	6–74	14 (22)^§^	9, 10, 11, 12, 16
Aleman et al., 2021 [57]	*RPE65*-LCA patients and healthy controls	7–18	3 (3)	7, 13
Jacobson et al., 2021 [23]	*GUCY2D*-LCA	7-37 visit 1 12-44 visit 2	10 (10)	7, 7, 11, 12, 13, 14 visit 1 12, 14, 16, 16 visit 2
Sumaroka et al., 2019 [105]	*CEP290*-LCA, *CEP290*-RD, *NPHP5*-LCA	7–30	18 (19)	7, 12, 12, 13, 13, 14, 15, 16, 17
Zobor et al., 2017 [62]	*CNGA3* incomplete and complete achromatopsia	7–56	27 (36)	No patients < 18 achieved FST
Testa et al., 2021 [33]	Biallelic *RPE65* mutations	8, 9	2 (2)	8, 9
Russell et al., 2022 [17]	*CEP290*-LCA	8–44	11 (11)	8, 10, 14, 15, 16
Cideciyan et al., 2019 [16]	*CEP290*-LCA	8–44	10 (10)	8, 10, 15, 16
Maguire et al., 2009 [40]	*RPE65*-LCA VN Ph I trial	8–44	7 (12)^‖^	8, 8, 10
Miraldi Utz et al., 2018 [18]	*TRPM1*-cCSNB	9–15	7 (7)	9, 9, 10, 11, 14, 15
Collison et al., 2015 [48]	*CEP290*-LCA	9–39	5 (6)	9
Roman et al., 2005 [1]	Retinal degeneration (RP, LCA, Usher syndrome type I and II, BBS, choroideremia, CORD), maculopathy (Stargardt, AD macular degeneration, early-onset macular drusen) and healthy controls	9–81	146 (158) achromatic 125 (158) chromatic 12 (12) healthy controls	Not individually reported
Roman et al., 2007 [2]	Retinal degeneration and healthy controls	9–83	61 (61) patients 9 (9) healthy controls	Not individually reported
Ngo et al., 2023 [73]	Retinitis pigmentosa (*BBS1, CLRN1, DHDDS, PDE6A, PDE6B, PROM1, RP1, SCAPER, TULP1, USH1C, USH2A, PRPF31, PRPH2, RP2)*	9–86	21 (21)	9, 12, 16, 17, 17
Sahel et al., 2021 [91]	*RPE65*-LCA	9-31	2 (8)	Not individually reported
Jacobson et al., 2012 [10]	*RPE65-*LCA VN Ph I dose escalation 3-year follow-up	11–30	15 (15)	11, 11, 15, 15, 16, 17
Bennett et al., 2016 [52]	*RPE65*-LCA VN Ph I follow-on trial	11–46	10 (11)	11, 12, 14, 14
Smirnov et al., 2022 [92]	*RBP4* retinal dystrophy	12	1 (1)	12
Krishnan et al., 2020 [63]	*CEP290*-LCA or *NPHP5*-LCA Healthy controls	12–33 22-26 healthy controls	6 (6) 4 (4) healthy controls	12, 14, 15, 16
Stunkel et al., 2018 [93]	*GUCY2D*-LCA	13–52	3 (5)	13, 14, 17
Cideciyan et al., 2021 [56]	*CEP290*-LCA	14	1 (1)	14
Stingl et al., 2021 [37]	*RPE65*-LCA	14–36	5 (5)	14
Ghazi et al., 2016 [14]	*MERTK* mutations	14–52	6 (6)	14
Birch et al., 2020 [22]	*USH2A* or ARRP	Not mentioned, possibly young as 5	56 (80) USH2A 37 (47) ARRP	Not individually reported
Roman et al., 2022 [88]	*GUCY2D* and *CEP290*	15–47	7 (7)	Five patients aged between 10–19
Dimopoulos et al., 2018 [32]	Choroideremia	17–67	30 (30)	17

*Baseline FST testing was not available in one patient aged 4 at baseline, results only available at age 5 at Y1; †7 patients had no perception of light (NLP) vision; ‡ one eye only; §FST was not available at baseline for all patients, and two patients could not perform due to young age; ‖Two patients were not tested due to equipment unavailable.

**Fig. 6:**
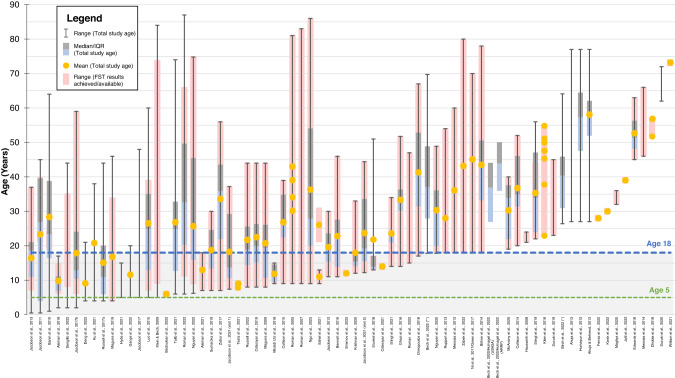
Summary graph of FST studies (*n* = 71)* ordered by total population age range, showing also individual ages of patients with FST results where available. The blue dashed line and grey area demarcates paediatric and adult patients. The green dashed line demarcates age 5 (pre-schoolage). *Nine studies were omitted from graph due to no available data on age.

A comprehensive summary figure (Fig. 6) visualises all FST studies by overall study age range, with age range for which FST results were achieved or available shown where reported. Demarcation lines show studies in which the overall population included patients aged <18 years (blue dashed line), or ≤5 years (green dashed line). From this figure, it can be appreciated that while there were 15 studies that included children aged 5 or under, only one study reported successful FST results in these younger children. These were two children with *RPE65* mutations in the VN Phase III clinical trial [12]. However, for the youngest child who was 4 years old at baseline, FST was achieved only at day 180 and year 1; results were missing at baseline and all other timepoints due to unreliable white light testing and procedural deviations [12, 13]. Subjective testing in infants aged less than 5 years of age can be limited by comprehension and/or cooperation, and there are currently no age-based modifications to the FST.

### Methodology and reporting

#### Nomenclature

**Fig. 7:**
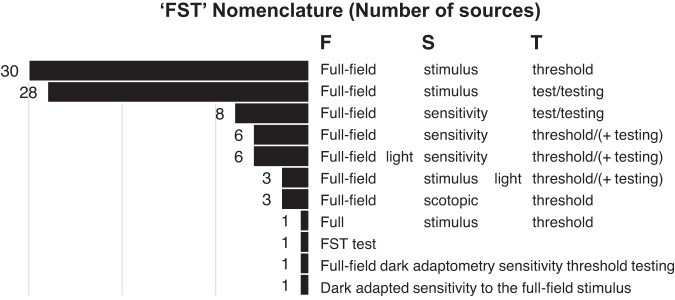
Variations in ‘FST’ full nomenclature used by sources (*N* = 80). Some sources use more than one form of nomenclature within the same text.

While commonly abbreviated to ‘FST’, there remains inconsistency in the full name of the test among sources. While the original studies [1–3] refer to the ‘full-field stimulus test’, other terminology used in literature and manufacturer materials include ‘full-field sensitivity test’, ‘full-field light sensitivity threshold’, ‘full-field scotopic threshold’ or similar variations and combinations of terms. Figure 7 shows these variations and number of sources in which they appear. Regardless of the shared abbreviation, inconsistency for the full name introduces additional ambiguity such as for keyword selection when performing systematic literature searching, or when sources may be translated or compared between centres. The World Health Organization have advised on the importance of standardising nomenclature of medical devices. Inconsistencies in the names of medical devices my cause confusion between types of devices, affect traceability, and adversely impact healthcare delivery [97].

#### Testing equipment

In 56 (70%) of 80 studies, FST was performed using hardware and software from Diagnosys LLC. This was often the Espion ColorDome™ LED full-field stimulator with the E2 or E3 desktop console Espion software version E6.49 (ref. [56]) or E6.59 (ref. [16]), and/or specific FST software. One group (2019; 2021) refer to a ‘thresholding algorithm built into a computer driven ERG system’ without mention of commercial software or hardware [35, 57]. The original iterations of the FST by Roman et al. were developed using a custom modified Zeiss-Humphrey perimeter [1, 2, 4]. Although the FST is also available on the MonCvONE-CR perimeter-based system by Metrovision [58], no studies have yet been published using this device for FST, although one study [59] mentions testing in one patient was performed using the Metrovision device before acquisition of the Diagnosys equipment. 20 studies (25%) did not specify the software or hardware used to record the FST responses.

The patient response interface for psychometric testing was mentioned only by 17 (21%) of the 80 studies, seven of which specified a ‘two button box’, ‘binary’ or ‘yes-no’ input [3, 27, 56, 59, 60], four studies reported ‘button box’, or ‘button-press’ [33, 48, 61, 62], and four ‘patient response’ [23, 31, 63], The review by Simunovic’s group [64] reported ‘target detection’. The perimeter-based methods with the original modified Humphrey or the MCvONE perimeters are known to use a single-button input [1–3, 58]. The remainder 78% of studies did not specify whether the response input was one or two-choice, and this cannot be otherwise inferred, since the commercialised Diagnosys device offers both one or two button input options, or alternatively the operator may also respond on behalf of patient’s verbal response [4, 65–67]. These methodological differences may affect comparability and interpretation of results in several ways. A single or dual-choice decision paradigm affects the complexity of the task (which may be particularly relevant when testing young children or patients with learning needs), and may alter the underlying psychophysical algorithm for threshold estimation. Moreover, using an operator response method may introduce additional response delay and require prolonging of the interstimulus interval (ISI), which could introduce fatigue or also alter the psychometric function.

#### Stimulus characteristics and thresholds

##### Colour

**Fig. 8:**
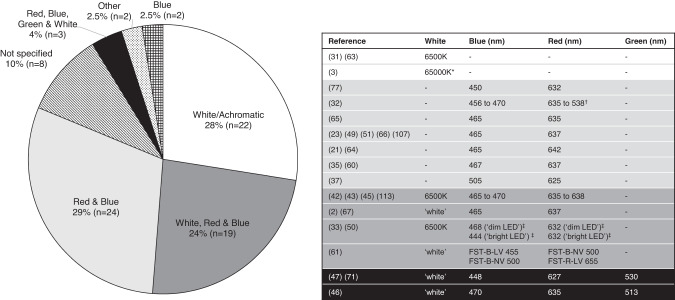
Colour and wavelength of flash stimuli used for full-field stimulus threshold testing (*n* = 80 studies). *Assumed to be typographical error (should be 6500 K). †Assumed to be typographical error (should be 638 nm). ‡‘dim’ LED < 0.01 cd∙s/m^2^, ‘bright’ LED > 0.01 cd∙s/m^2^.

Regarding the colour of stimuli, 22 (28%) of the 80 studies reported FST thresholds were obtained using white or achromatic ‘6500 K’ stimuli; 24 (29%) studies used red and blue LED stimuli (peak wavelength ranges were 632–642 nm and 450–465 nm, respectively); 19 (24%) used white, red and blue stimuli; and three referred to white red, blue and green (peak 513–530 nm) stimuli, although green light FST data were not used in analysis [46, 54, 68]. Two studies tested FST thresholds using blue stimuli only [9, 69], and stimulus colour was not specified in eight studies. Two studies were categorised as ‘other’ due to being a review [4] or post-hoc analysis of existing data [30] (Fig. 8).

Of the 51 studies that reported performing FST using chromatic stimuli, 34 cited the purpose was to delineate which photoreceptors mediated the FST response by using the difference in measured sensitivity to ‘blue’ (presumed rod) and ‘red’ (presumed cone) stimuli. Only 18 of these studies specified the blue-red difference criteria for distinguishing rod, cone or mixed photoreceptor mediation. Differences between blue-red FST ranged from ≥19 to 28 dB for rod-mediated thresholds (with more sensitive thresholds to blue compared to red stimuli), ≤0 to 10 dB for cone-mediation (similar sensitivity to blue and red stimuli) and values between these bounds indicating mixed rod-cone mediation (Table 4). The manufacturer information for the Metrovision FST suggests a difference between thresholds to blue and red stimuli of 19 dB characterises rod mediation [58].

**Table 4:** Differentiating rod and cone contributions to sensitivity thresholds tested using chromatic full-field stimuli.

Source	Patient population	Rod mediation	Cone mediation	Mixed mediation
Charlier et al., 2019 [58]	N/A [Commercial Information]	*“Difference of 19* *dB between the thresholds with the red and blue flashes”*		
Banin et al., 2010 [72] Jacobson et al., 2011 [24]	*RPE65*-LCA *AIPL1*-LCA	Blue-red difference ≥ 19.3 dB	Blue-red difference ≤ 3.1 dB	Blue-red difference between 3.1 and 19.3 dB
Stingl et al., 2019 [19]	*CRB1*-RP	Blue-red difference ≥ 20 dB	Blue-red difference <5 dB	
Birch et al., 2022 [30] Hufnagel et al., 2022 [80]	*USH2A-*Usher Syndrome and *USH2A*-ARRP	Blue-red difference ≥ 20 dB	Blue-red difference ≤10 dB (lower limit −30 dB)	
McAnany et al., 2021 [61]	X-linked retinoschisis	>2 log units more sensitive to the blue compared to the red stimulus		
Dimopoulos et al., 2018 [32] Nguyen et al., 2020 [51] Jacobson et al., 2021 [24] Talib et al., 2021 [59] Nguyen et al., 2022 [20]	Choroideremia *RPGR*-RP and *RPGR*-CORD *GUCY2D*-LCA *CRB1*-RP, *CRB1*-CORD, *CRB1* macular dystrophy *CRB1*-RP, *CRB1*-CORD, *CRB1* macular dystrophy	Blue-red difference > 22 dB	Blue-red difference <3 dB	Blue-red difference between 3 and 22 dB
Roman et al., 2007 [2]	Inherited retinal disease, healthy controls	Blue-red difference ≥ 23.3 dB, consistent with 25.4 dB expected for the theoretical consideration of both stimuli being perceived by scotopic (rod) vision		
Roman et al., 2022 [4]	Inherited retinal disease (LCA, RP, Usher, CORD, BBS, choroideremia, BCM, achromatopsia, Stargardt disease)	Blue-red difference expected ≥ 25.4 dB		
Roman et al., 2005 [1] *(NB: 12* *dB added to measured sensitivity levels of blue stimulus to equate energy to that of red stimulus)*	Inherited retinal disease (RP, LCA, Usher I and II, BBS, choroideremia, CORD, Stargardt disease, macular degeneration, early-onset macular drusen), healthy controls	Blue-red difference ≥ 28 dB	Blue-red difference ≤12 dB	Blue-red difference between 12 and 28 dB
Jacobson et al., 2017 [83]	*GUCY2D*-LCA	Blue-red difference statistically greater than zero (difference > 0.4 log units)	Light adapted threshold blue minus red difference <2.14 log units	
Collison et al., 2014 [27]	*ABCA4*-Stargardt Disease	Red-blue difference ≤ 2.2 dB	Average response to red stimuli during the cone plateau (5–10 min after bleach)	
Collison et al., 2015 [48]	*CEP290*-LCA	Red-blue difference ≤ 2.2 dB	Red-blue difference 0 dB	Red-blue difference between 0.1 and 2.1 dB
Zabek et al., 2022 [63]	RP (various including *USH2A, EYS, RPGR, CRB1, DHX38, RHO, RP1, POMGNT1, PHYH, FAM161A, RP9*)	White FST threshold below -30 dB (based on Birch et al, 2020)		

Birch’s group [22] compared white, red and blue thresholds in the RUSH2A study to phenotype *USH2A*-associated retinopathy. They noted patients with ≥ 20 dB difference between blue and red thresholds mostly had white thresholds less than -30 dB. This led to the proposal that any white FST threshold dimmer than –30 dB is rod-mediated (reference level 0 dB = 0.1 cd/m^2^) though some exceptions can be noted in those with long-standing USH2A disease. Zabek’s group adopted this classification of rod-mediation for their white FST thresholds tested in RP patients (reference level 0 dB = 0.1 cd·sm^2^) [60].

Stimulus presentation order was often not specified for two-colour testing. When three or more colours were tested, one study group specified an order of blue-red-white [46, 68], and another used blue-white-red [33]. Two groups recommended red-blue-white [41, 42, 44], three groups used white-red-blue [2, 27, 48, 51], and for the remainder of studies order was not specified. In the original paper by Roman’s group [1], the stimulus testing sequence was: white, white, blue, red, white, blue, red, white.

##### Temporal presentation and response time or ISI

For stimulus temporal characteristics, the Diagnosys software allows customisation options of ‘flash’, ‘pulse’ or ‘blink’ [65, 67], while the Metrovision system presents a ‘flash’ every 3 s [58]. 51 (64%) of the 80 studies did not specify stimulus type or duration. Of those that did, 12 studies reported using a 200 millisecond (ms) flash stimulus, eight used a 4 ms flash, three reported a ‘brief full-field flash’, while six stated simply ‘flashes’. The interstimulus interval (ISI) or response time-window was specified by Klein and Birch [3] and Ahuja’s group [70] as 5 s, though the Diagnosys*FST* software enables customisation of ISI between 1 and 9999 ms [65, 67]. An ‘unconstrained response window’ was used in the sepofarsen clinical trials, when patients with *CEP290-*LCA were tested using a commercial binary thresholding algorithm. Though one patient with a substantial treatment response (P11) was assessed further using chromatic FST under dark and light-adapted conditions using a 4/2 dB staircase and two response reversals with a ‘limited’ time window, reported to minimise false-positive responses (i.e. any responses not synchronised with stimulus presentation) [16, 56]. Roman et al. [4] suggest the ISI may need to be prolonged for severely affected patients. For inter-session duration, Roman [2] and Ghazi’s [14] groups suggested a ‘short’ pause between threshold determinations to avoid fatigue, Messias’ group [41, 42] specified 5 min interval between sessions for their patients with diabetic retinopathy, while ‘no timeout’ was specified in the study with RP patients by Zabek et al. [60]. The majority (81%) of studies did not specify ISI or duration between threshold determinations or between sessions.

This omission in reporting stimulus temporal characteristics again complicates interpretation and data comparability. It was reported that two patients with *CEP290*-LCA (P7 and P9) in the sepofarsen clinical trial were erroneously tested using a 4 ms stimulus at several timepoints instead of the protocol-specified 200 ms [16, 17]. In the interim report, one of these patients (P7) was tested under both conditions at 3 months and appeared to show a ~1 log unit better sensitivity to the blue (but not to the red) stimuli for the longer stimulus presentation [16]. This is noteworthy since 1 log unit (or 10 dB) improvement in FST sensitivity is suggested to constitute a clinically significant post-treatment change in *RPE65* patients [11, 33, 71]. In the phase 1b/2 report the authors decided to exclude the data from this patient and impute the data for the second patient (P9) who was erroneously tested [17]. The temporal characteristics of the light stimulus (and hence also the ISI) may have consequences for the psychometric function, as well as the conversion of the units of light stimulus between the absolute (cd∙s/m^2^, cd/m^2^) or relative luminance units (dB).

#### Testing Strategy

The original versions of the FST developed by Roman’s group from a modified perimeter used a staircase algorithm initially that varied stimuli in 4 dB steps and then in 2 dB steps with each reversal (4-2-2), with the final threshold estimate as the luminance last seen by the patient [1]. The commercially available Diagnosys*FST* programme describes a seen/not seen strategy and explores the detection of stimuli within a 10 dB range of a selected starting luminance. If the threshold is not found within this 10 dB range, the software shifts the area of exploration to a 10 dB range up or down according to a proprietary algorithm which includes no stimulus ‘catch’ trials until a threshold is reached. It should be noted that although described in some texts as ‘forced-choice’ [3], this yes/no strategy is distinct from and has less control against response variation bias compared to true two-alternative forced choice methods where a choice is made between two versions of concurrent or sequential stimuli. The final threshold in the Diagnosys*FST* programme is calculated as the midpoint of the frequency of seeing curve generated using a two parameter Weibull function, accounting for false positives (‘Error Blanks’) and false negatives (‘Error Max’) [3, 64, 65, 67]. The Metrovision MonCvONE perimeter employs an 8-4-2-1 staircase sequence, with occasional tests for patient reliability using no stimulus ‘catch’ trials [58].

Reporting of the psychophysical strategy is inconsistent among the 80 included studies. Of 12 studies that mentioned a ‘staircase’ strategy, 11 referred to using the ‘4-2-2’ strategy despite testing using the commercialised system. One study used a 5-2.5-2.5 staircase [72] and another stated that ‘16 reversals were required for convergence’ [70]. 31 studies did not provide details of FST methodology or strategy but cited previous references to published methodology, most often that of [1–3]. Test strategy was not specified or referenced in 19 studies.

For estimation of the final threshold, 12 studies reported this to be determined as the median or 50% probability of detection on the sigmoidal psychometric function, while eight specifically referenced the built-in two parameter Weibull function. Twelve other studies reported that the final threshold was taken as the average of multiple measurements per colour stimuli per eye (usually 2, 3, or 10 measurements per stimuli). Roman et al. [4] advise 6 measurements per session to be the most appropriate compromise for estimating variance and avoiding fatigue – with two sessions of 6 measurements being sufficiently powered (98%) to detect a 5 dB FST change on two-sample 2-sided *t-*test at 5% significance.

#### Patient preparation

Where pupillary dilation was specified (22 studies; 28%), this was most frequently done with 1% tropicamide and 2.5% phenylephrine. Testing was nearly always performed monocularly (usually with other eye patched), apart from three studies that performed binocular testing [51, 69, 72] and 15 studies where this was not specified.

In total, 60 (75%) of the 80 total studies reported FST performed under dark adapted (DA) conditions and 9 studies mentioned light-adapted testing, with or without DA testing. Only four studies specified DA methodology and six studies detailed light adaptation (Table 5). For DA methodology, the Diagnosys*FST* commercial information advises only to ‘set the patient in a dark room and start the adaptation timer’ [65, 67] and allows a choice of duration. The shortest DA time reported was 20 min [19, 73], and the Metrovision*FST* also references 20 min of DA [58]. The longest DA duration was ‘overnight’ in a natural history study in *RLBP1*-RP patients [74].

**Table 5:** Dark adaptation (DA) and light adaptation (LA) methods for full-field stimulus testing, where available, and ordered by duration of DA duration.

Source	Patient population	DA duration	DA methodology	LA methodology
Diagnosys LLC, 2019 [67]	N/A	Not specified	“Set the patient in a dark room and start the adaptation timer”	N/A
Stingl et al., 2019 [19] Charlier 2019 [58] Ngo et al., 2023 [73]	*CRB1*-RP N/A Mixed RP	20 min	Not specified	N/A
Ruppert et al., 2013 [89] Messias et al., 2014 [41] Messias et al., 2015 [42] Dhoble, Hess & Venkatesh, 2018 [44]	RP ( + controls) Proliferative DR ( + controls) Proliferative DR Non-proliferative DR, proliferative DR	25 min	Not specified	N/A
Klein & Birch 2009 [3] Humayun et al., 2012 [81] Ahuja & Behrend, 2013 [50] Ahuja et al., 2013 [70] Messias et al., 2013 [31] Collison et al., 2014 [27] Collinson et al., 2015 [48] Zobor et al., 2017 [62] Klein et al., 2018 [85] McAnany et al., 2020 [61] Hyde et al., 2021 [21] Nguyen et al., 2022 [20]	Mixed RP Mixed RP, LCA, choroideremia ‘End-stage’ RP ‘End-stage’ RP RP Stargardt disease ( + controls) *CEP290*-LCA ( + controls) *CNGA3* Achromatopsia Mixed RP ( + controls) XLRS ( + controls) *CNNM4* Jalili syndrome *CRB1*-RP, *CRB1*-CORD, *CRB1* macular dystrophy	30 min	Not specified	N/A 5 min of a full-field bleach (3.1 log cd/m^2^ 6500 K achromatic full-field), which bleached ~67% of rhodopsin N/A
Dimopoulos et al., 2018 [32] Talib et al., 2021 [59] Zabek et al., 2022 [60]	Choroideremia ( + controls) *CRB1*-RP, *CRB1*-CORD, *CRB1* macular dystrophy Mixed RP	≥ 30 min	Dim-lit room and patch Not specified Not specified	5 min bleach using a 3.0 log cd/m2 6,500 K achromatic full-field stimulus (previously shown to bleach 67% of available rhodopsin molecules) N/A N/A
Sengillo et al., 2022 [54]	Biallelic *RPE65* retinopathy	25–40 min	Not specified	N/A
Bennett et al., 2016 [52] Testa et al., 2021 [33]	*RPE65-*LCA *RPE65* retinopathy	40 min	Not specified	N/A
Jolly et al., 2016 [25] Jolly et al., 2017 [26] Edwards et al., 2018 [15] Miraldi Utz et al., 2018 [18] Suzuki et al., 2019 [46] Suzuki et al., 2020 [68]	Choroideremia ( + controls) Choroideremia ( + controls) Mixed RP *TRPM1*-cCSNB Healthy controls RP ( + controls)	45 min	Not specified	N/A
Roman et al., 2005 [1] Roman et al., 2007 [2] Ghazi et al., 2016 [14]	Mixed retinal degeneration, maculopathy ( + controls) Mixed retinal degeneration ( + controls) *MERTK*-RP	≥ 45 min ≥ 45 min > 45 min	Contralateral eye ‘triple patched’ with adhesive eye patch, black opaque photographic tape, black eye patch with elastic band, all lights off	N/A
Hauswirth et al., 2008 [9]	*RPE65*-LCA	1 h	Not specified	N/A
Aleman et al., 2018 [35]	*RDH12*-LCA, *RDH21*-EORD ( + controls)	45 min + 15–20 min after pupillometry and dilation	Not specified	N/A
Roman et al., 2022 [4]	Inherited retinal disease (LCA, RP, Usher, CORD, BBS, choroideremia, BCM, achromatopsia, Stargardts)	45 min or 2 h	Light exposures (whether by indirect ophthalmoscopy, fundus photography or other procedures) were rescheduled in the protocol for times during the visit that followed dark-adapted testing. Patients were instructed to wear sunglasses from waking and keep them on before coming to the clinic; not to gaze into lights (e.g. their cell phones). They were made aware of the negative effect of light exposure on their ability to sense the dimmest lights under dark-adapted conditions.	Light adaptation was performed with four white flashes (each 3.88 log scot-cd.s.m^−2^ and 3.58 log phot-cd.s.m^−2^) presented serially within seconds while the subject gazed straight, right, left, and up, in, order to adapt as much of the retinal area as possible
Ni et al., 2017 [74]	*RLBP1*-RP	Overnight	Overnight dark adaptation	Bleach
Jacobson et al., 2017 [83]	*GUCY2D*-LCA	Not specified	Not specified	Steady 2.7 logTd background
Jacobson et al., 2021 [82]	*GUCY2D*-LCA	Not specified	Not specified	Steady white 10 phot cd.m^−2^ background

*CORD* cone-rod dystrophy, *cCSNB* complete congenital stationary night blindness, *DA* dark adaptation, *DR* diabetic retinopathy, *EORD* early-onset retinal degeneration, *LCA* Leber congenial amaurosis, *RP* retinitis pigmentosa, *XLRS* X-linked retinoschisis.

Patients were dark-adapted for 25 min in four studies, 30 or ‘at least 30’ min in fifteen studies, 40 min in two studies, 45 or ‘at least 45’ min in nine studies, and 1 h in one study (Table 5). Sengillo et al. [54] reported 25–40 min DA, while Aleman et al. [35] reported a DA duration of >45 min plus additional 15–20 min for pupillary dilation. There was no appreciable rationale for choice of DA duration (no apparent associations with patient population, VA, age etc), though Stingl’s group [19] chose 20 min based on the minimum ISCEV recommendations for scotopic full-field ERG at the time [98]. 22 studies where dark-adapted thresholds were tested did not specify DA duration. Williams et al. [45] performed chromatic FST without DA, but acknowledged that this limited their ability to interpret their blue FST data as isolated rod-function responses.

In the phase 1 VN gene therapy dose escalation trial, chromatic FST thresholds were tested after both a’standard’ DA time of <2 h and ‘extended’ DA time of >3 h, due to the authors’ prior observation of prolonged rod (but not cone) kinetics on scotopic perimetry in treated *RPE65*-LCA eyes [99]. Only extended DA blue flashes responses were reported, while extended DA red flash responses were reported only if cone-mediated [10]. The short-term phase 1 trial reported 1 h DA for blue FST [9], which shortened to 40 min DA for chromatic and achromatic stimuli in the phase 1 follow-on trial report [52]. The later VN clinical trial publications did not specify DA method or duration. In their recent review, the original FST developers advise a 45 min protocol only if values are not different to those tested under 2 h DA [4].

### Interpretation of FST results

#### Units

Interstudy comparison and interpretation of FST data is complicated by the variability in units used to represent threshold values of luminance. 51 studies (64%) reported in decibels (dB, a ratio-based scaling of luminance units) while 19 studies (24%) reported in log units of luminance i.e. log(cd∙s/m^2^) or log(cd/m^2^). Some studies used a combination of both dB and log units [24, 69] while other units in the literature included log scotopic Trolands [47], LogMAR [43], and a percentage of the ‘*maximum threshold needed to elicit the FST response’*, which the authors state was done to ‘minimise the ceiling effect and produce a meaningful figure’ [15]. Four studies did not report any units (Table 6).

**Table 6:** Units used in the reporting and measuring of retinal sensitivity luminance thresholds in full-field stimulus threshold testing sources.

Source	Count (Total *N* = 85)	Units	dB reference	
Maguire et al., 2009 [40] Banin et al., 2010 [72] Bittner et al., 2014 [77] Messias et al., 2014 [41] Bennett et al., 2016 [52] Ghazi et al., 2016 [14] Jolly et al., 2016 [25] Jolly et al., 2017 [26] Zobor et al., 2017 [62] Aleman et al., 2018 [35] Klein et al., 2018 [85] Miraldi Utz et al., 2018 [18] Suzuki et al., 2019 [46] Nguyen et al., 2020 [51] Suzuki et al., 2020 [68] Aleman et al., 2021 [57] Ku et al., 2021 [55] Roman et al., 2022 [4] Sahel et al., 2021 [91] Sengillo et al., 2022 [54] Testa et al., 2021 [33] Chung et al., 2021 [78] Birch et al., 2022 [30] Simunovic et al., 2022 [64] Hufnagel et al., 2022 [80] Smirnov et al., 2022 [92] Kwak et al., 2022 [86]	27	dB	Not specified	
Roman et al., 2005 [1] Messias et al., 2013 [31] Dimopoulos et al., 2018 [32] Stingl et al., 2019 [19] McAnany et al., 2020 [61] Hyde et al., 2021 [21] William et al., 2022 [45]	7		0 dB = 0.01 cd∙s/m^2^	
Stingl et al., 2021 [37] Nguyen et al., 2022 [20] Ngo et al., 2023 [73]	3		0 dB = 0.01 cd/m^2^	
Klein & Birch, 2009 [3] Collison et al., 2014 [27] Collison et al., 2015 [48] Stunkel et al., 2018 [93] Talib et al., 2021 [59] Jalil et al., 2022 [84] Zabek et al., 2022 [60]	7		0 dB = 0.1 cd∙s/m^2^	
Birch et al., 2020 [22]	1		0 dB = 0.1 cd/m^2^	
Ahuja & Behrend, 2013 [50] Ahuja et al., 2013 [70] Dagnelie et al., 2010 [79] Diagnosys LLC, 2019 [66] Diagnosys LLC, 2019 [67]	5		0 dB = 3 cd∙s/m^2^	
Charlier, 2019 [58]	1		0 dB = 318 photopic cd/m^2^ 111 dB = 2.54 ×10^−9^ cd/m^2^	
Jacobson et al., 2021 [23]	1		0 dB = -0.57 log/(photcd/m^2^)	
Roman et al., 2007 [2]	1		0 dB = 3.7 cd/m^2^	
Sumaroka et al., 2019 [105]			“Converted to HFA sensitivity scales” (0 dB = 12 phot-cd/m^2^ for red for dark-adapted static perimetry)	
Testa et al., 2021 [33] Diagnosys, Birch & Aleman, 2020 [29]	2		dB[cd∙s/m^2^] (no 0 dB reference given)	
Jacobson et al., 2011 [24] Bedoukian et al., 2022 [69]	2	dB and log units		
Hauswirth et al., 2008 [9] Jacobson et al., 2012 [10] Jacobson et al., 2013 [82] Jacobson et al., 2017 [49] Jacobson et al., 2017 [83] Ni et al., 2017 [74] Wang et al., 2020 [94]	7	Log units		log units
Russell et al., 2017 [11] Russell et al., 2017 [90] Maguire et al., 2019 [12] Magliyah et al., 2020 [71] Bennett et al., 2021 [76] Gange et al., 2022 [53] Deng et al., 2021 [34] Leroy et al., 2022 [87]	8			log (cd∙s/m^2^)
Cideciyan et al., 2019 [16] Krishnan et al., 2020 [63] Cideciyan et al., 2021 [56] Russell et al., 2022 [17]	4			log (cd/m^2^)
Roman et al., 2022 [88]	1			log units (0 dB = -3.8 scot-cd/m^2^)
Luo et al., 2015 [47]	1			log scotopic trolands
Ferraz Sallum et al., 2022 [43]	1			LogMAR
Edwards et al., 2018 [15]	1	% (of maximum threshold needed to elicit the FST response)		
Humayun et al, 2012 [81] Ruppert et al., 2013 [89] Messias et al., 2015 [43] Diagnosys LLC, 2016 [65] Dhoble, Hess & Venkatesh, 2018 [44]	5	Not specified		

*cd* candelas, *dB* decibels, *s* seconds, *FST* full-field stimulus threshold, *m* metres.

For reference, the definitions of common photometric units are provided in Appendix Table 5. and can be found in the ISCEV calibration guidelines [100]. Candelas per square metre (cd/m^2^) is the International System of Units (SI) standard unit of luminance. This measures light emitted from a source surface per unit area, and is used for display screens or ganzfeld background luminance. For brief flashes of light, such as the stimuli used in ERG and FST testing, ‘flash strength’ is given as candela-seconds per square metre (cd·s/m^2^). This weights luminance by the flash duration to account for the temporal integration of the visual system. The MLMT, the mobility test used for the primary endpoint of the VN Phase III clinical trials, measures the levels of the various light conditions in units of illuminance (lux) [12]. Illuminance is a measure of the amount of light falling onto or received by a given surface area, and decreases as the distance between the surface and the light source increases. Trolands are calculated by multiplying luminance of the stimulus by pupillary area, to give an estimate of the effective stimulus at the retina.

Photometric measures are matched to the spectral sensitivity of the light-adapted eye (peaking at 555 nm). Rod stimuli may be more accurately measured in scotopic units that correct for the spectral sensitivity of the dark-adapted eye (peaking ~500 nm), typically using scotopic filter over a photometer, however such photometer filters are not widely available. Hence, the ISCEV calibration standards recommend using photopic units but note that for a short wavelength rod flash, a xenon strobe of 2–3 photopic cd·sm^2^ is equivalent to 4 scotopic cd·sm^2^ [100].

For the studies reporting in dB, there was further variation in the 0 dB reference point used for converting between relative (dB) and absolute luminance (i.e. cd∙s/m^2^) units. Although it is possible to customise this conversion base in the Diagnosys*FST* programme setup and interconvert between units within the software, the variability in published literature may also point to inaccuracies of reporting. For example, Klein and Birch [3] used a 0 dB = 0.1 cd∙s/m^2^ reference, reportedly equivalent to 25 cd/m^2^ presented for 4 ms. Nguyen’s group [20] reported their 0 dB set to 0.01 cd/m^2^, but also stated that this was equivalent to 25 cd/m^2^ presented for 4 ms, despite there being no time component in their reported units. An earlier study with the same patient cohort set the 0 dB reference to 0.1 cd∙s/m^2^, but both studies state the same ‘healthy’ control reference value of -53 dB (assumed to be rod mediated and derived from [1–3]) despite the apparent difference in log unit scaling [59].

There were 26 studies that reported FST results in dB but did not specify a 0 dB reference point. This has implications not only on data comparability between centres, comparison with control reference data, but also on interpretation of clinically significant change. For example, a result of -60 dB would correspond to –8 log units if 0 dB was set to 0.01 cd∙s/m^2^, but to -7 log units if 0 dB was set to 0.1 cd∙s/m^2^. This means without specification of unit parameters, a patient tested at two different time-points could be erroneously reported as having a -1 log unit clinically significant improvement in retinal sensitivity due to ambiguity of the reference scales used.

For post-treatment *RPE65* patients, a clinically significant post-treatment change in retinal sensitivity measured on FST is considered to be >10 dB or >1 log unit improvement in white light threshold (averaged across both eyes) from baseline [11, 12, 52]. Nonetheless, this still represents a relative rather than absolute change in retinal sensitivity (i.e. the retina being sensitive to a ten-fold decrease in stimulus luminance). Test-retest variability was most frequently between 1–3 dB or 0.1 to 0.3 log units, often calculated as the 2 standard deviations from the mean threshold, across 15 studies with available data.

#### Reference FST data from healthy controls

**Fig. 9:**
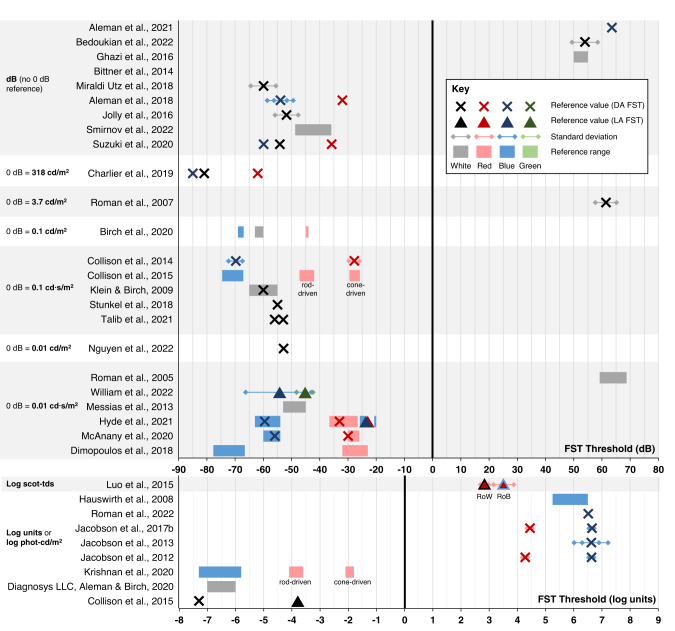
Summary figure showing control reference data for full-field stimulus testing where available. Studies are grouped according to units and 0 dB reference scaling. Note that data are plotted in their original reported values of each study, and so may not be directly comparable between studies that use different 0 dB scales. DA dark adaptation, FST full-field stimulus threshold, LA light adaptation.

Reference FST threshold values based on results from visually healthy controls were available in 32 studies, either numerically reported in the text or represented on figures. Direct comparison of reference values between studies is challenging due to the variability in units used (dB, log phot-cd/m^2^ or log scot-tds) and different conventions in positive or negative scaling of luminance parameters. Moreover, some studies used a reference range and others a mean value with standard deviation or standard error. It may also be considered that reference values may localise to the control population available to each centre. The reference range for Diagnosys*FST* are suggested to be –6 to –7 log units for white flashes [29], while for the Metrovision-*FST*, responses from a healthy control participant measured –85 dB, –62 dB and –81 dB for white, red and blue flashes respectively, with 0 dB equal to 318 cd/m^2^ [58]. Figure 9 presents available healthy control reference data from included sources, with values as originally reported in the text.

## Discussion and conclusions

Having demonstrated utility in pivotal gene therapy clinical trials, FST has emerged as an increasingly adopted visual functional outcome measure both in clinical research settings and the eye clinic. The need for wider harmonisation of methodology and reporting is clear [4], and this scoping review has not only summarised characteristics of current FST practice but also highlighted instances where interstudy practice variability may preclude data comparability, interpretation, and meaningful clinical follow-up. Additionally, despite indication for better therapeutic potential for treating IRD patients at a younger age, FST has rarely been achievable in children aged ≤5 years, with protocol modification and age-matched reference data an underdeveloped research area in this topic.

### Considerations for methodological standardisation

Although the commercialised FST programmes offer flexibility in test customisation, the lack of formalised standard guidance is reflected by the numerous current permutations in test parameters such as patient interface (e.g. one or two-button response paradigm, verbal or button-press response); stimulus parameters (colour/wavelength, order of presentation, temporal characteristics, response time window, number of reversals etc); threshold calculation strategy (rod/cone mediation criteria; method of calculating final threshold); and patient preparation (method and duration of dark or light adaptation, patient instruction, mydriasis, monocular or binocular testing etc).

Each element of variation introduces additional ambiguity or potential alterations to the underlying psychometric function. This affects comparability, reproducibility and interpretation of data between studies which may have significant consequences for future multicentre clinical trials, evidence synthesis, assessment of quality or agreement between centres, or establishment of regional or national references databases. Investigators and standardisation stakeholders may wish to consider that some test parameters may be specific to the purpose and clinical population being investigated, and generate context-based guidance modifications accordingly.

### Considerations for reporting and interpretation

A notable finding from this scoping review is the high proportion of studies that currently omit crucial elements of FST methodology in their reporting, such as characteristics of the light stimulus (4 ms or 200 ms) or 0 dB reference level. This may be due to a need for concise methodology in papers, and sometimes studies sometimes simply reference the original publications of [1, 3] while clearly using an individualised protocol. Arguably, unit differences may be arbitrary if the clinical aim is to compare sensitivity change from baseline for individuals, provided parameters remain consistent for the same patient. Nevertheless, these reporting inconsistencies limit reliable post-hoc analyses such as for systematic evidence synthesis or meta-analyses. As FST becomes more favoured as a visual functional endpoint in clinical trials (such as in NCT04516369), investigators may wish to consider the Delphi approaches to the generation of core outcome sets [101, 102] to assess and prioritise minimum elements of FST methodology that are the most important to report.

### What is a clinically significant change in FST

Relatedly, it must be considered that the clinically significant 10 dB or 1 log unit change suggested by the *RPE65* clinical trials represent relative improvement proportional to the baseline residual sensitivity. A ten-fold increase in FST threshold from -1 to -2 log units represents improvement by 0.09 cd∙s/m^2^, but from -4 to -5 log units is a 0.00009 cd∙s/m^2^ improvement (using 0 dB = 0.1 cd∙s/m^2^). This requires patients with a higher baseline threshold value to demonstrate a larger step-change in absolute luminance sensitivity from baseline to constitute improvement. This also has implications for infants or patients with severely low vision, in whom it may be challenging obtain an accurate baseline if at all, as demonstrated in [12] and several other studies where FST results were omitted due to unreliable performance or lack of baseline comparison.

Interpretation is additionally complicated by lack of consensus on the source of remnant vision i.e., whether FST is a summative global response or mediated by the most sensitive retinal loci. There is the question of whether intrinsic photosensitive retinal ganglion cells may also contribute to visual perception to flash stimuli in the absence of outer retina [4, 48, 103]. Pupillometry studies have suggested that longer duration (1000 ms) and brighter white or blue (2.4–2.6 log cd/ms^2^) stimuli favours a melanopsin-mediated transient pupillary light reflex (TPLR) whereas TPLR under shorter flash durations (e.g. 100 ms) is likely more driven by remnant outer retina [75, 104].

Clarification of structure-function associations and FST response origin will be important not only for understanding of disease pathophysiology, but also for predicting treatment potential from retinal structure or other factors [105]. In treated *RPE65* eyes, Stingl et al. [37] showed evidence of clinically relevant retinotopic rod function improvement using FST, DA chromatic perimetry and chromatic pupil campimetry, but also found younger age to be a major predictor of degree of functional photoreceptor rescue. Conversely, Gange’s group [53] found despite progressive perifoveal chorioretinal atrophy in 18 eyes of 10 treated *RPE65* patients, average FST improvement remained consistent at 3 log units, VA was not significantly altered, and VF improvement was broadly stable on 1 year follow-up. Notably, 9 of the 10 patients described with treatment-related atrophy were aged <18 years. These findings further elaborate the significance of structure-function and paediatric-specific considerations in the treatment and post-treatment monitoring of *RPE65* retinal dystrophy, which may be applicable also to other IRDs.

### Considerations for adaptation for specific populations

It is readily appreciated that psychophysical testing in younger children can be constrained by cooperation, understanding and cognitive/motor demand, particularly for sequentially presented stimuli requiring a subjective response. Studies have found that lapse rate (incorrect response to ‘catch trials’) for simple psychophysical tasks can be 19% in neurotypically developing children compared to 5% in adults [106]. These factors may be further amplified in children with early visual impairment and/or atypical sensory processing [107, 108]. Non-compliance with scotopic testing may be compounded by unfamiliar clinical settings and the need for mydriasis and lengthy dark adaptation. Accurate paediatric psychophysical measurement requires a careful balance between efficiency (e.g. more reversals reduce variability, but longer procedures may result in higher lapse rate) and the need for procedural standardisation to ensure comparability between participants [106].

Some strategies to account for inattentional bias and high lapse rate include indexing the lapse rate through statistical methods such as checking consistency of reversal points [106], or estimating threshold through post-hoc fitting of psychometric function on QUEST-based procedures rather than taking the staircase reversal average [109]. Lapses may also be physiologically monitored through recording of eye movements or posture [106, 110] or using neurophysiological correlates [111]. Furthermore, investigators may opt to develop novel strategies to minimise likelihood of lapses through improving task engagement by children, such as through gamified approaches e.g. [112, 113].

Although commercialised automation of the FST enables a single threshold estimation to be completed relatively quickly (3 min for Metrovision and 2–8 min for *Diagnosys*FST), recommendations to perform several runs per stimuli per eye for a more reliable average (Roman et al. [4] recommend 6 repeats) with 5 min breaks between trials [41, 42] can prolong test time. Intuitive strategies to improve test efficiency include selecting an appropriate starting luminance based on manual pre-testing or previous estimates [4, 65], though this is not yet standardised or clearly reported (e.g. how many dB above the pre-test estimate is appropriate to set the starting value). In younger children or patients with learning needs, it may be that investigators consider alternative methods of estimating starting luminance based on visual behaviour gauged through preferential looking techniques or spatial frequency limits [114, 115].

Nevertheless, the largest contributor to total test duration is dark adaptation (DA) time, of which there is no current consensus. The range in included studies is 20 min to 3 h (or overnight in one instance), with the most frequently reported being 30 min. The original test developers recommend 45 min DA, provided values are not different from those tested under 2 h DA [4]. Anecdotally, for young *RPE65* patients with additional learning or communicational needs and their families 45 min DA is challenging and sometimes distressing. Alternatives to provide a physiologically meaningful measure are needed for these instances. There appear to be ongoing investigation into chromatic FST performed under ‘mesopic’ conditions (0.1 cd/m^2^ background illumination) without pupil dilation or dark adaptation [116].

With FST being a test of absolute visual thresholds. Roman et al. [4] caution that rod adaptation kinetics may be particularly prolonged in newly treated *RPE65* eyes [99]. It may be that investigators develop condition-specific DA protocols to accommodate this phenomenon in *RPE65* patients, while opting for alternative appropriate DA durations for baseline testing or in other retinal conditions, using data derived from literature or empirical research. It might be further argued that for purposes of monitoring or assaying light perception in low vision patients, the priority is to standardise conditions to facilitate longitudinal comparison against baseline or a control reference, regardless of where the test point may fall on the patient’s individual DA curve. Furthermore, as with other tests of visual function, FST is rarely performed in isolation and should be interpreted within the constellation of other structural and functional measures [64, 117].

### Future perspectives

FST emerged among several novel specialised approaches to assay vision at the absolute thresholds, driven by the need to develop outcome measures for low vision patients to assess therapeutic benefit in gene therapy clinical trials. As FST can be delivered using existing ganzfeld hardware or a modified perimeter, it has become a widely available and increasingly favoured supplementary measure of retinal sensitivity for both research and clinical monitoring purposes.

This scoping review has summarised some key areas of current variability in FST practice, discussed some impacts of non-standardisation, and highlighted the under-researched space of paediatric-specific considerations in this field. The next steps are for stakeholders to conduct further evidence synthesis or empirical research to establish consensus on a set of coherent methodological guidance (which may need to be specific to the type of retinal condition under investigation), protocol modifications for specialist populations and importantly a minimum set of elements for reporting (for example, 0 dB reference level, response task, and key stimulus parameters such as colour, duration and ISI, length of dark adaptation). In parallel to this, agreement on criteria for quality assurance, stimulus calibration and establishment of local or regional age-matched reference databases will also be crucial to enhance reliability and rigour of measurement [100, 118].

### Supplementary information

Appendix Tables

## Data Availability

All data generated and/or analysed during the current study are available from the corresponding author(s) on reasonable request.
