# Supplementary material for: Full-field stimulus threshold testing: a scoping review of current practice
Source: Eye (Lond). 2023 Jul 13;38(1):33–53. doi: 10.1038/s41433-023-02636-3 (PMC10764876; doi:10.1038/s41433-023-02636-3)
Supplement: Supplementary file 1 — Appendix Tables [file 41433_2023_2636_MOESM1_ESM.docx]

# **Appendix Table 1.** Full search query for each electronic database

| Database | Search query |
| --- | --- |
| The Cochrane Library  (Title, Abstract, Keywords) | (final AND (dark-adapt* OR "night vision" OR rod OR scotopic) AND threshold*)  OR (FST AND full-field)  OR (“full-field stimulus threshold”  OR "scotopic sensitivity tester"  OR "scotopic sensitivity testing"  OR "whole-field scotopic") |
| Embase  (All Fields + Text) | ((final AND (dark-adapt* OR "night vision" OR rod OR scotopic) AND threshold* AND retin*)) OR ((FST AND full-field AND retin*)) OR (("full-field stimul*" AND threshold*)) OR "scotopic sensitivity test*" OR "scotopic sensitivity threshold*" OR "scotopic visual threshold*" OR "whole-field scotopic" OR "wholefield scotopic" |
| Pubmed  (All Fields) | (final AND (dark-adapt* OR "night vision" OR rod OR scotopic) AND final AND threshold*)  OR (FST AND full-field)  OR "full-field stimulus threshold"  OR "scotopic sensitivity tester"  OR "scotopic sensitivity testing"  OR "whole-field scotopic"  OR "wholefield scotopic" |
| Scopus | TITLE-ABS-KEY (final AND (dark-adapt* OR "night vision" OR rod OR scotopic) AND threshold* AND retin*)  OR (FST AND full-field AND retin*)  OR ("full-field stimul* threshold*"  OR "scotopic sensitivity test*"  OR "scotopic sensitivity threshold*"  OR “whole-field scotopic”  OR “wholefield scotopic”) |
| Web Of Science (All Databases)   - Web Of Science Core Collection - Biosis Citation Index - KCI-Korean Journal Database - Medline, Russian Science - Citation Index - Scielo Citation Index | TS=((final AND (dark-adapt* OR “night vision” OR rod OR scotopic) AND threshold* AND retin*)  OR (FST AND full-field AND retin*)  OR ("full-field stimul* threshold*"  OR "scotopic sensitivity test*"  OR "scotopic sensitivity threshold*"  OR “whole-field scotopic”  OR “wholefield scotopic”)) |
| Grey Literature Sources:   - British Library E-Theses Online Service (Ethos) - Google / Google Scholar - Opengrey - WHO International Clinical Trials Registry Platform (WHO ICTRP) - Commercial/Educational Material from Manufacturers | Keywords such as *‘full-field stimulus threshold’*, *‘FST’, “final scotopic threshold”, “whole-field scotopic retinal sensitivity”*, *“scotopic sensitivity tester”* |

# **Appendix Table 2.** Initial inclusion criteria in Population, Concept, Context format

|  | Include | Exclude |
| --- | --- | --- |
| Population | - Human participants of any age and level of vision | - Animal/non-human only studies |
| Concept | - Psychophysical test that measures retinal sensitivity thresholds using a full-field stimulus and an adaptive psychophysical methodology e.g.   - Full-field stimulus threshold (FST) testing,   - Whole-field scotopic retinal sensitivity testing (SST-1)   - Final scotopic threshold from dark adaptometry | - Dark adaptometry without final scotopic threshold reported - Tests not employing a full-field stimulus |
| Context | - Clinical and/or research settings where FST has been performed and/or reported on in human participants. | - Animal/biochemical only settings |
| Types of Evidence Sources | - All empirical, theoretical and conceptual peer-reviewed publications and grey literature where methodology for measurement of scotopic retinal threshold using full-field stimulation has been sufficiently described. | - Testing is mentioned but no further methodological or reporting detail |

# **Appendix Table 3.** Data extraction form categories and subcategories

|  | Categories | Subcategories |
| --- | --- | --- |
| Key source characteristics | Authors |  |
|  | Journal/platform |  |
|  | Publication year |  |
|  | City/State |  |
|  | Country |  |
|  | Original language |  |
|  | Type of source/study | Type |
|  |  | Registration number |
|  | Key objectives/ purpose |  |
|  | Purpose of using FST specifically |  |
|  | Declared interests |  |
|  | Source of funding/ support |  |
| Participants | Number of participants (n) | Total |
|  |  | By study group/medical indication |
|  |  | By genetic variant |
|  | Number of eyes (n) | Total |
|  |  | By study group/medical indication |
|  | Age (years) |  |
|  | Protocol follow-up |  |
|  | Disease duration (y) |  |
|  | Number (%) male |  |
|  | Visual acuity /level | Original units |
|  |  | LogMAR (SD) |
| Concept (Test) characteristics | Nomenclature |  |
|  | Test system | Hardware and software |
|  |  | Manufacturer |
|  | Patient interface |  |
|  | Stimulus set-up parameters | Colour/wavelength of stimulus |
|  |  | Order of stimulus presentation |
|  |  | Testing strategy |
|  |  | Dynamic stimulus range |
|  |  | Stimulus temporal characteristics (flash/pulse/blink) |
|  |  | Response cue |
|  |  | Response window duration |
|  |  | Units |
|  | Patient preparation | Dilation |
|  |  | OD/OS/BEO |
|  |  | Light adaptation method |
|  |  | Dark adaptation method |
|  |  | Dark adaptation duration |
|  |  | Patient instruction |
|  | Starting luminance strength | Starting luminance |
|  |  | Method to determine starting luminance |
|  | No. of trials per eye |  |
|  | Quality specification |  |
|  | Threshold calculation strategy | Method for calculating final threshold |
|  |  | Method for distinguishing type of threshold |
|  |  | Reference value (healthy controls) |
|  | Other ophthalmic tests performed |  |
| Findings | Statistics | Methods |
|  |  | Software |
|  |  | Significance set (p<) |
|  | FST outcomes | Number of participants with reportable FST obtained: number (eyes) |
|  |  | Thresholds |
|  |  | Within-session repeatability |
|  |  | Inter-session repeatability |
|  |  | Clinical significance definition |
|  |  | Relationship with other tests or parameters |
|  | Key FST conclusions |  |
|  | Key study conclusions |  |

# **Appendix Table 4.** List of scoping review included sources (N=85) with first author, year of publication, title and journal or platform.

| **First Author** | **Year** | **Journal/platform** |
| --- | --- | --- |
| Roman et al. | 2005 | *Experimental Eye Research* |
| Roman et al. | 2007 | *Physiological Measurement* |
| Hauswirth et al. | 2008 | *Human Gene Therapy* |
| Klein & Birch | 2009 | *Documenta Ophthalmologica* |
| Maguire et al. | 2009 | *The Lancet* |
| Banin et al. | 2010 | *Human Gene Therapy* |
| Dagnelie et al. | 2010 | *Investigative Ophthalmology & Visual Science* [abstract] |
| Jacobson et al. | 2011 | *Investigative Ophthalmology & Visual Science* |
| Jacobson et al. | 2012 | *Archives of Ophthalmology (Chicago, Ill.: 1960)* |
| Humayun et al. | 2012 | *Ophthalmology* |
| Messias et al. | 2013 | *Documenta Ophthalmologica* |
| Ahuja & Behrend | 2013 | *Progress in Retinal and Eye Research* |
| Ahuja et al. | 2013 | *Translational Vision Science & Technology* |
| Jacobson et al. | 2013 | *Human Molecular Genetics* |
| Ruppert et al. | 2013 | *Investigative Ophthalmology & Visual Science* [abstract] |
| Collison et al. | 2014 | *Retina* |
| Messias et al. | 2014 | *Investigative Ophthalmology & Visual Science* [abstract] |
| Bittner et al. | 2014 | *Clinical and Experimental Optometry* |
| Messias et al. | 2015 | *Investigative Ophthalmology & Visual Science* [abstract] |
| Luo et al. | 2015 | *PLoS ONE* |
| Collison et al. | 2015 | *Investigative Ophthalmology & Visual Science* |
| Ghazi et al. | 2016 | *Human Genetics* |
| Jolly et al. | 2016 | *Investigative Ophthalmology & Visual Science* [abstract] |
| Bennett et al. | 2016 | *The Lancet* |
| Diagnosys LLC | 2016 | *N/A* |
| Russell et al. | 2017 | *The Lancet* |
| Jolly et al. | 2017 | *BriSCEV 2016 Conference* [poster] |
| Jacobson et al. | 2017 | *Investigative Ophthalmology & Visual Science* |
| Zobor et al. | 2017 | *Visual Neuroscience* |
| Ni et al. | 2017 | *Investigative Ophthalmology & Visual Science* [abstract] |
| Jacobson et al. | 2017 | *American Journal of Ophthalmology* |
| Russell et al. | 2017 | *Investigative Ophthalmology & Visual Science* [abstract] |
| Edwards et al. | 2018 | *Ophthalmology* |
| Miraldi Utz et al. | 2018 | *JAMA Ophthalmology* |
| Dimopoulos et al. | 2018 | *Retina* |
| Aleman et al. | 2018 | *Investigative Ophthalmology & Visual Science* |
| Dhoble, Hess & Venkatesh | 2018 | *Documenta Ophthalmologica* [abstract] |
| Klein et al. | 2018 | *Investigative Ophthalmology & Visual Science* [abstract] |
| Stunkel et al. | 2018 | *American Journal of Ophthalmology* |
| Maguire et al. | 2019 | *Ophthalmology* |
| Cideciyan et al. | 2019 | *Nature Medicine* |
| Stingl et al. | 2019 | *Translational Vision Science & Technology* |
| Suzuki et al. | 2019 | *Documenta Ophthalmologica* [abstract] |
| Charlier | 2019 | *Metrovision.fr* |
| Diagnosys LLC | 2019 | *N/A* |
| Diagnosys LLC | 2019 | *N/A* |
| Sumaroka et al. | 2019 | *Investigative Ophthalmology & Visual Science* |
| Birch et al. | 2020 | *Translational Vision Science and Technology* |
| Diagnosys LLC, Aleman & Birch | 2020 | *N/A* |
| Nguyen et al | 2020 | *International Journal of Molecular Sciences* |
| McAnany et al. | 2020 | *Investigative Ophthalmology & Visual Science* |
| Krishnan et al. | 2020 | *Vision Research* |
| Suzuki et al. | 2020 | *Documenta Ophthalmologica* [abstract] |
| Magliyah et al. | 2020 | *Documenta Ophthalmologica* |
| Wang et al. | 2020 | *Orphanet Journal of Rare Diseases* |
| Hyde et al. | 2021 | *Ophthalmic Genetics* |
| Jacobson et al. | 2021 | *International Journal of Molecular Sciences* |
| Testa et al. | 2021 | *Translational Vision Science and Technology* |
| Stingl et al. | 2021 | *The British Journal of Ophthalmology* |
| Ku et al. | 2021 | *Investigative Ophthalmology & Visual Science* [abstract] |
| Cideciyan et al. | 2021 | *Nature Medicine* |
| Aleman et al. | 2021 | *Clinical Ophthalmology* |
| Talib et al. | 2021 | *Acta Ophthalmologica* |
| Bennett et al. | 2021 | *Investigative Ophthalmology & Visual Science* [abstract] |
| Chung et al. | 2021 | *Clinical Experimental Ophthalmology* [abstract] |
| Sahel et al. | 2021 | *American Journal of Ophthalmology* |
| Roman et al. | 2022 | *Progress in Retinal and Eye Research* |
| Russell et al. | 2022 | *Nature Medicine* |
| Nguyen et al. | 2022 | *American Journal of Ophthalmology* |
| Birch et al. | 2022 | *Investigative Ophthalmology & Visual Science* |
| Deng et al. | 2022 | *Graefe's Archive for Clinical and Experimental Ophthalmology* |
| Ferraz Sallum et al. | 2022 | *Ophthalmic Genetics* |
| William et al. | 2022 | *Clinical Ophthalmology* |
| Gange et al. | 2022 | *Ophthalmology Retina* |
| Sengillo et al. | 2022 | *Ophthalmology Retina* |
| Zabek et al. | 2022 | *Graefe's Archive for Clinical and Experimental Ophthalmology* |
| Simunovic et al. | 2022 | *Survey of Ophthalmology* |
| Bedoukian et al. | 2022 | *Ophthalmic Genetics* |
| Hufnagel et al. | 2022 | *Wiley Human Mutation* |
| Jalil et al. | 2022 | *Eye (London)* |
| Kwak, Kim & Byeon | 2022 | *Yonsei Medical Journal* |
| Leroy et al. | 2022 | *Archives of Disease in Childhood* [abstract] |
| Roman et al. | 2022 | *BMC Ophthalmology* |
| Smirnov et al. | 2022 | *International Journal of Molecular Sciences* |
| Ngo et al. | 2023 | *American Journal of Ophthalmology* |

**Appendix Table 5.** Definition of photometric units and examples of usage. Adapted from definitions by the Système Internationale (SI) and International Society for Clinical Electrophysiology of Vision (ISCEV). Conversion between candela per square metre and older luminance units can be found in the ISCEV Calibration Standard Guidelines (80). Note that the Troland does not directly convert to other units since it is calculated based on pupil size. *ERG, electroretinography; FST, full-field stimulus threshold test; MLMT, multi-luminance mobility test; VEP, visual evoked potential.*

| Standard photometric values | | | | | Other values | |
| --- | --- | --- | --- | --- | --- | --- |
| Quantity | **Luminous flux** | **Luminous intensity** | **Luminance** | **Luminance exitance** (exiting)  **Illuminance** (incoming) | **Time-integrated luminance** (or ‘flash strength’) | **‘Retinal Illuminance’** |
| SI Unit | Lumen, lm | Candela (lumen per steradian), cd = lm/sr | Candela per square metre, cd/m^2^ | Lumen per square metre, lm/m^2^ or lux, lx = lm/m^2^ | Candela second per square metre, cd·s/m^2^ | Troland, td  cd/m^2^ x pupil area mm^2^ |
| Property | *Non-directional* | *Directional* | *Directional and per unit area* | *Non-directional and per unit area* | *Directional, per unit area and time-integrated* | *Directional, per unit area and scaled by pupil size* |
| Illustration | 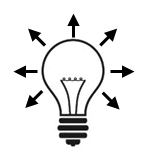 | 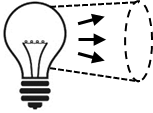 | 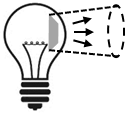 | 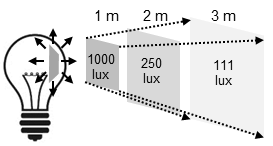 | 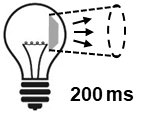 | 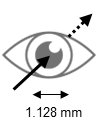 |
| Examples of uses |  | A common candle traditionally emits light with ~1 cd | Visual display screens, letter charts, ganzfeld background | Light condition levels in the MLMT | Brief flashes of light e.g. stimuli typically used for flash ERG, flash VEP, or FST | Pupillometry, some handheld ERG devices also measure pupil size |
| Definition | Amount of light emitted by a light source, as perceived by the human eye (standardised on 555 nm peak).* | Amount of light per unit solid angle emitted by a source in a given direction | Amount of light per unit solid angle per unit area emitted from an extended source (or reemitted from a reflective surface), in a given direction. | Luminance exitance: Amount of light *emitted* by a surface of given area.  Illuminance: Amount of light *falling on* or *being received* *by* a surface of given area. Illuminance decreases as distance from the source increases. | Luminance of a flash stimulus weighted by the flash duration (to account for the temporal integration by neuronal visual pathways) | Approximate amount of retinal illumination when a 1 cd/m^2^ stimulus is viewed through a pupillary area of 1 mm^2^ (diameter 1.128 mm). |
| *The lumen is defined for photopic cone vision and does not adequately measure visibility under low light levels <0.01 cd/m^2^ when rods mediate vision. Scotopic units can be measured using a filter (such as ILL700 SED033/ZCIE/W) over the photometer which matches the wavelength responsivity of the eye under dark adapted conditions (typically peaking ~500 nm). Since these filters are not widely available, the ISCEV Calibration Standards suggest the use of photopic units. | | | | | | |
